# Developing non-invasive 3D quantificational imaging for intelligent coconut analysis system with X-ray

**DOI:** 10.1186/s13007-023-01002-4

**Published:** 2023-03-09

**Authors:** Yu Zhang, Qianfan Liu, Jing Chen, Chengxu Sun, Shenghuang Lin, Hongxing Cao, Zhaolin Xiao, Mengxing Huang

**Affiliations:** 1 School of Computer Science and Technology, Hainan University, Haikou, China; 2 Radiology department, Central South University Xiangya School of Medicine Affiliated Haikou Hospital, Haikou, China; 3 Coconut Research Institute, Chinese Academy of Tropical Agricultural Sciences, Wenchang, China; 4 School of Computer Science and Engineering, Xi’an University of Technology, XI’an, China; 5 School of Information and Communication Engineering, Hainan University, Haikou, China

**Keywords:** Intelligent coconut analysis, Non-invasive, Point cloud, Quantitative imaging model

## Abstract

**Background:**

As one of the largest drupes in the world, the coconut has a special multilayered structure and a seed development process that is not yet fully understood. On the one hand, the special structure of the coconut pericarp prevents the development of external damage to the coconut fruit, and on the other hand, the thickness of the coconut shell makes it difficult to observe the development of bacteria inside it. In addition, coconut takes about 1 year to progress from pollination to maturity. During the long development process, coconut development is vulnerable to natural disasters, cold waves, typhoons, etc. Therefore, nondestructive observation of the internal development process remains a highly important and challenging task. In this study, We proposed an intelligent system for building a three-dimensional (3D) quantitative imaging model of coconut fruit using Computed Tomography (CT) images. Cross-sectional images of coconut fruit were obtained by spiral CT scanning. Then a point cloud model was built by extracting 3D coordinate data and RGB values. The point cloud model was denoised using the cluster denoising method. Finally, a 3D quantitative model of a coconut fruit was established.

**Results:**

The innovations of this work are as follows. 1) Using CT scans, we obtained a total of 37,950 non-destructive internal growth change maps of various types of coconuts to establish a coconut data set called “CCID”, which provides powerful graphical data support for coconut research. 2) Based on this data set, we built a coconut intelligence system. By inputting a batch of coconut images into a 3D point cloud map, the internal structure information can be ascertained, the entire contour can be drawn and rendered according to need, and the long diameter, short diameter and volume of the required structure can be obtained. We maintained quantitative observation on a batch of local Hainan coconuts for more than 3 months. With 40 coconuts as test cases, the high accuracy of the model generated by the system is proven. The system has a good application value and broad popularization prospects in the cultivation and optimization of coconut fruit.

**Conclusion:**

The evaluation results show that the 3D quantitative imaging model has high accuracy in capturing the internal development process of coconut fruits. The system can effectively assist growers in internal developmental observations and in structural data acquisition from coconut, thus providing decision-making support for improving the cultivation conditions of coconuts.

## Introduction

**Fig. 1:**
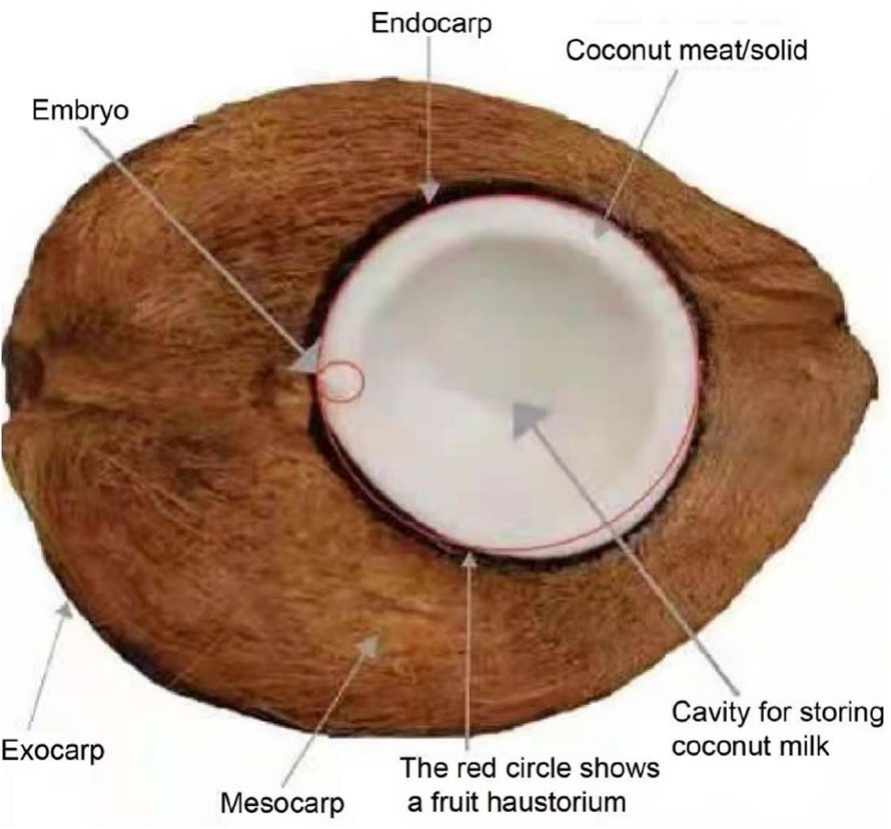
The sectional structure of coconut fruit

Coconut palms (*Cocos nucijera* L.) generate oil-bearing seeds which are essential for the production of vegetable oil in the marketplace. The coconut fruit is a fibrous drupe, not a nut. A drupe is a simple fleshy fruit containing one seed, derived from one ovary of a flower. A thin skin covers the outermost layer of the ovary wall. The coconut has a thick, fibrous middle layer of the ovary wall. The inner layer of the ovary wall is hard and contains one seed. A seed nut planted in a coconut nursery is a fruit. So the fruit wall consists of the exocarp, mesocarp, endocarp (coconut husk), solid endosperm (coconut meat), liquid endosperm (coconut water) and embryo (Fig. 1). The coconut fruit has evolved to have three layers of peel: the exocarp is leathery, thin and smooth, providing effective protection against external moisture; the mesocarp is fibrous, and protects the fruit from breaking when dropped; the peel is bony, hard and not easily deformed, and can effectively protect the embryo and endosperm of the coconut.

The palm has distinctive characteristics emerge throughout the germination and seedling growth processes. The seeds have a tiny embryo and a large quantity of endosperm. The distal section of the embryo expands in size to form a haustorium, which remains within the seed and expands significantly when the endosperm disappears. When the haustorium develops, the outer surface, which is closely attached to the endosperm, extends significantly. A numerous, minute, and protuberant structure is particularly developed on the attached surface. The undulating structure is pale yellow in color. Endosperm breakdown products saturate the surface and lodge in the troughs caused by invaginations. The epithelium, the haustorium’s outermost layer, is composed of rectangular cells. During haustorium development, cells of the inner parenchyma progressively grow in size.

Coconut palms are divided into two types based on their physical traits and breeding habits: tall coconut palms and dwarf coconut palms. Tall coconuts cross-pollinate spontaneously, and the populations exhibit various degrees of heterozygozity [1]. Dwarf coconut, on the other hand, is naturally self-pollinating and exhibits typical morphological traits, including dwarf height, a thin trunk, a smaller crown, and small-sized nuts with relatively low copra content. Because of the coconut fruit’s unique structure and growth, cultivators find it difficult to examine the inside, making non-destructive inspections even more crucial to ensuring natural growth. To overcome this issue an innovative radiographic imaging method known as X-ray computed tomography allows for non-destructive, non-invasive three-dimensional (3D) imaging at higher resolutions as well as utilizing a mathematical technique, researchers have examined the internal structure of the same fruit from several perspectives to determine the fruit’s three-dimensional volume [2, 3].

The images were obtained by X-ray from the Computed Tomography (CT) machine has been proposed in different fruit. For instance, Cantre et al. [4] utilized CT to examine the microstructure of epidermal and sub-epidermal tissue in kiwi fruit, whereas Ting et al. [5] used CT to analyze the microstructures of various apple cultivars. On the other hand, CT were used to quantify and describe the internal structure of intact pomegranate fruit [6]. Few research were carried out to evaluate internal fruit disorders such mealiness in pears [7], internal browning in apples [8, 9], watercore in apples [10], and cracked stones in Japanese plums, CT has been used several times [11]. The results of all this research have contributed to an understanding of how internal disorders impact fruit microstructure and how these abnormalities might be prevented. More recently, CT was used to investigate the distribution of thermo-physical characteristics such porosity and thermal conductivity in Japanese apricot [12]. Janssen et al. [13] investigated the 3-dimensional (3D) pore structure of ‘Braeburn’ apples employing CT.

Therefore, we propose a CT-based non-invasive 3D quantitative imaging system for coconuts. As a consequence, in this research, we used the coconut as the object and developed a method for constructing a 3D model of the coconut using CT scan images. The purpose of this paper is to investigate the internal stratified physiological structure of coconut fruit while identifying its growth pattern through quantitative imaging model. The established model of coconut is highly accurate and intuitive, forming a reliable non-destructive evaluation method, which can improve the screening rate of effective coconut fruits, reduce the presence of bad fruits, and improve the breeding efficiency of seedlings. It has a high application value in the field of coconut production.

## Materials and methods

**Fig. 2:**
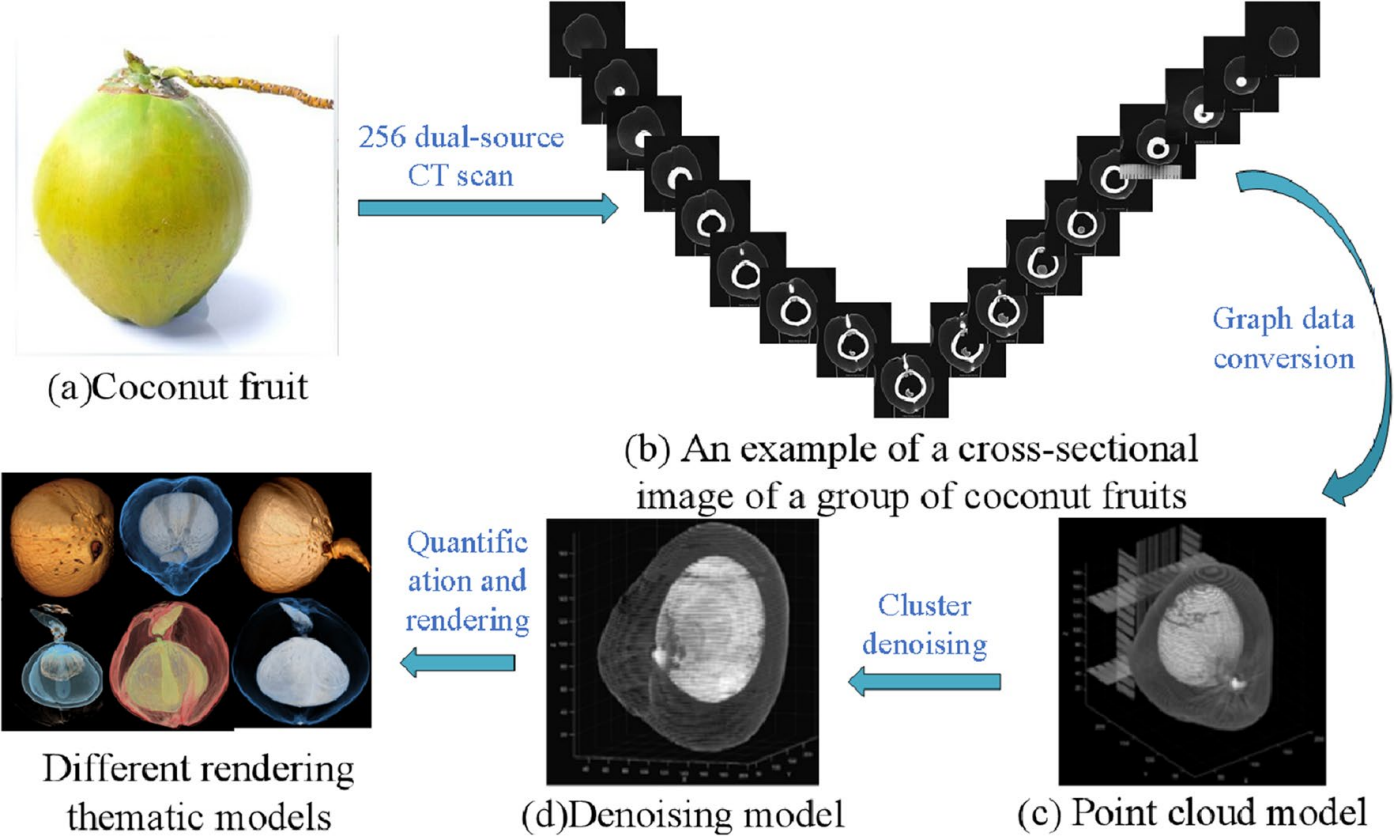
An overview of coconuts reconstruction via 3D Quantificational Imaging

The process of establishing a non-invasive 3D quantitative imaging system using CT to as a method for monitoring coconut fruit development (Fig. 2). We use CT axial scanning to obtain a set of cross-sectional images of coconut fruit [14]. Then the coordinates of the points in the cross-section image group are extracted, and the corresponding 3D point cloud images are generated by array transformation and stacking. The graph is then processed by K-nearest neighbor denoising. After fitting the outline of the outer surface, the system shows a complete three-dimensional quantitative model of the coconut fruit. Initially, CT coronal scanning of the coconut fruit was used to produce a series of cross-sectional images of the coconut fruit, which was then used to construct the point cloud model by extracting the 3D coordinate data of the cross-sectional image group and the RGB value data of the point. After that, a 3D quantitative model of coconut fruit was developed.

### Cross-sectional image acquisition of the coconut fruit by CT coronal scan

In order to continuously observe the growth process of the coconut fruit, we first number the sample coconut fruit, then mark the posture position of the coconut fruit to ensure that it was in a fixed position in each scan. Then we used CT scanning to obtain the corresponding coronal scan image of each coconut fruit (Fig. 2b).

**Fig. 3:**
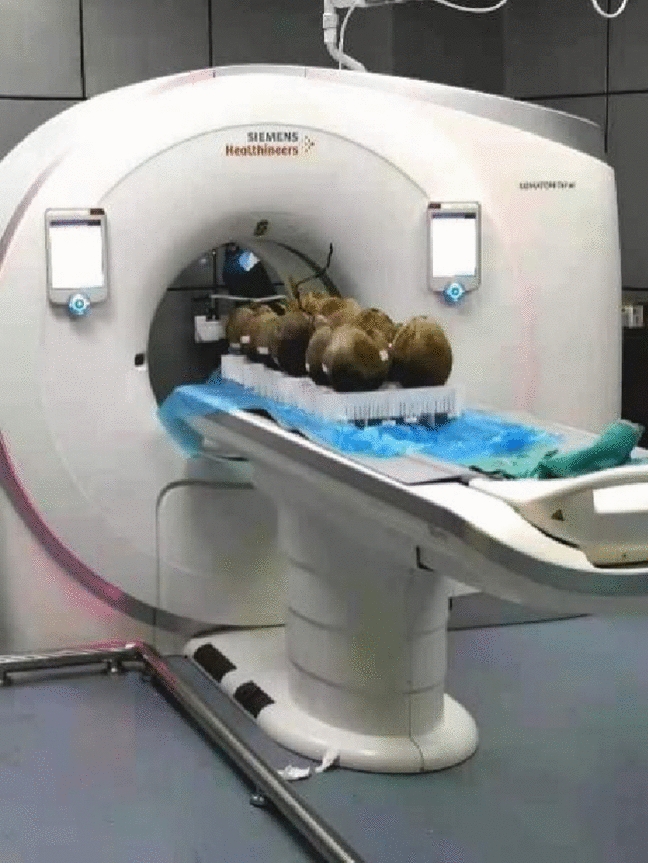
CT machine scans coconut samples

The coconuts were placed on a plastic shelf. Text and arrow markers were used so that the orientation of the image remained consistent throughout the cycle. The image was obtained using a 256 dual source CT scanner (Scanning instrument: SIEMENS SOMATOM Definition Flash) with the following parameters: tube voltage: 120 kV, tube current: 250 mas, temperature: 24 $^\circ$C, Humidity: 50%. Use a set of coconut samples were scanned by a CT scanner(Fig. 3).

### Point cloud model

After obtaining the CT image of the coconut, the next goal was to convert them into a point cloud model [15]. In MATLAB (MATLAB version: R2020b), the program first calls the ID of the target coconut, which is compressed and reconstructed into an array of 512$*$512$*$6. According to the Definition 1, the 6 columns are $\langle x,y,z,r,g,b\rangle$. The converted data of each image are stacked, the points are drawn according to the corresponding coordinate information values, and the 3D point cloud is displayed (Fig. 2c).

#### **Definition 1**

A coconut fruit point cloud model is a tuple $< X,Y,Z,R,G,B>$, where *X*, *Y* and *Z* are the 3D coordinate data whereas, *R*, *G* and *B* are the the Red, Green and Blue value respectively.

1. Pre-processing: The CT images derived from the direct scan of the CT machine are large, so they are compressed into 512$*$512 for ease of processing.
2. Image thresholding segmentation: recognition extracts the coconut part and distinguishes the effective information domain from the background of the CT images.

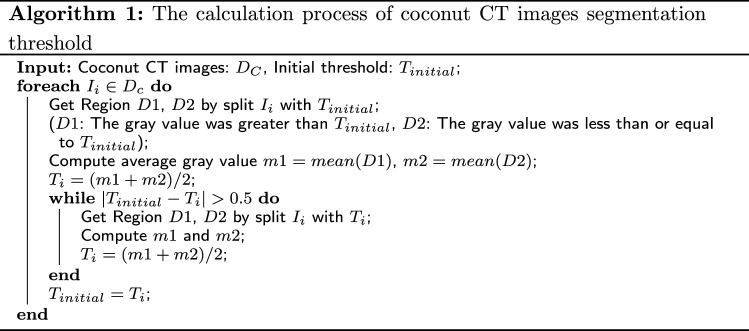


The algorithm 1 computes the segmentation threshold for coconut CT images. We implement the algorithm with Matlab. First, an initial threshold T is calculated based on manual experience, and then the image is segmented with T. D1 comprises all pixel components with gray values larger than T, whereas D2 includes all pixel components with gray values less than or equal to T. The average gray value m1 and m2 in each region are then computed, and the average value is used as the next threshold, and so on until the optimal threshold is established.

The equation for determining images after thresholding are

1 $$\begin{aligned} g(x,y)= {\left\{ \begin{array}{ll} 1&{} f(x,y)>T\\ 0&{} f(x,y)<T \end{array}\right. } \end{aligned}$$

where *f*(*x*.*y*) represents the original image, *T* is the finalized threshold, pixels marked with 1 correspond to objects and those marked with 0 are considered as background and invalid information.

(3) Conversion and merging of point cloud map data:
  - The reshape function was used to reconstruct a specific matrix *B* to fit the dimension of the coconut point cloud, $B = reshape(A,size)$, where A represents its own array of grayscale maps,and size was the reconstructed array dimension. In our work the value of size was 6.
  - Repelem was a function that creates an array of repeating elements *b*. $b = repelem(A,r_1,\ldots ,rN)$ repeats each element of *A* by $r_1,\ldots ,r_N$, returning an array where each element of $r_1,\ldots ,r_N$ must be a scalar or vector with the same length as *A* in the corresponding dimension [16]. This was due to the fact that in the 3D coordinate system, one quantity was the same on each of the *x*, *y*, and *z* dimensions.
  - Repmat was used to build a large matrix containing multiple repetition matrices *B*, $B = repmat(A,r)$, using the row vector r to specify the repetition scheme. $B = repmat(A,[mnp...])B = repmat(A,n)$, with the contents of *A* stacked in $(M\times N)$ matrix *B*. The size of the *B* matrix was determined by $M\times N$ and the contents of the *A* matrix, with the same coordinates on the same axis, matching the 3D array created $B = repmat(A,n)$ returns an array containing n copies of *A* in its row dimension and column dimension. when *A* is a matrix, the size of *B* is size $(A)\times n$. $B = repmat(A,r_1,\ldots ,r_N)$, specifying a list of scalars $r_1,\ldots ,r_N$ that describe how the copies of *A* are arranged in each dimension. When *A* has *N* dimensions, the size of *B* is $size(A)\times [r_1, \ldots , r_N]$.
  - PCmerge was used to merge multiple point clouds. By the previous method, we get an array ofpoint clouds for each layer, and then we need to merge and stack them to form a complete 3-dimensional point cloud map. This was expressed as: ptCloudOut = pcmerge(ptCloudA, ptCloudB, ptCloudC..., ptCloudN, gridStep); ptCloud refers to the point cloud data points, N = the number of coconut point cloud layers created, the merged point cloud was returned using the box grid filter, gridStep specifies the size of the filter’s 3D box, and in addition, increases the size of gridStep when there were not enough resources to build a large fine-grained grid.

The merged point cloud was returned as a point cloud object, and the PCmerge function calculates the axial bounding box of the overlapping area between the two point clouds. The bounding box was divided into grid boxes of the size specified by gridStep. The points in each grid box are merged by averaging their positions, colors, and normals. Points outside the overlapping area are not affected according to the storage order of the image itself [17]. Because the coconut was placed in a fixed direction according to the orientation markings made during each scan, there was no need be concerned with the image alignment generated by CT. The first pixel of the previous image must correspond to the first pixel of the next image, and after repeated tests, the layer spacing of each image was set to 0.225 cm as the best. The point cloud data are merged in this manner, thus constructing the coconut model. The 3D point cloud model of the coconut was constructed (Fig. 5a).

### Model optimization

After the initial point cloud model was built, there were two main problems: irrelevant information and the existence of image noise, and the discrete discontinuity of points on the outer surface. These two factors would affect the effectiveness and accuracy of the model, so we carried out denoising and surface fitting to improve the quality of the overall model [18].

#### Denoising method

We used K-Nearest Neighbor (KNN) noise reduction method to remove the noise points and set the threshold to 0.13, determine the distance between the center point and the surrounding 50 points, take the average value and compare it with the threshold value, and categorize the points contained within the threshold distance as the valid information class; the remaining classes were deleted. Then for a new sample *x*, the *k* points in the training set that are nearest neighbors to *x* are found [19]. How is this nearest neighbor measured? We use $L_p$ distance to measure it. The $L_p$ distance of two vectors $x_1$, $x_2$ can be defined as $L_p\left( {x}_{1},{x}_{2}\right) =\left( \sum _{{i}=1}^{{n}}\left| {x}_{1}^{{i}}-{x}_{2}^{{i}}\right| ^{{p}}\right) ^{\frac{1}{{p}}}$, next, find the class with the highest number of occurrences of the category among the *k* points and assign *x* to this class,expressed in a mathematical formula is

$$y = \arg \max c_j P_I (y_i = c_j), \quad i = 1,2,\ldots ,N;j = 1,2,\ldots ,K$$,

where i takes values from 1 to N, representing the number of samples in the training set, and j takes values from 1 to k, representing the number of categories in the training set. i is the indicator function, meaning that I is 1 when $y = c$, and I is 0 otherwise [20]. Thus obtain the effect after denoising (Fig. 5b).

**Fig. 4:**
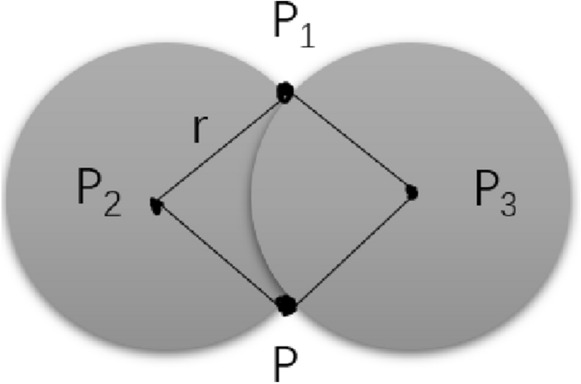
Definition of the connection between two points

#### Surface reconstruction and mesh quantization

The 3D model constructed by the point cloud method has the effect of displaying too many discrete points, which is not conducive to the integrity and attractiveness of the model. According to the relationship that defines the connection between two points (Fig. 4), we use the rolling ball method in order to construct the complete boundary information and calculate the associated volumes using integrals through the small grid thereby created.

1. For any point *p*, the radius of the rolling circle *r*, search all points within the distance 2*r* from the point *p* in the point cloud, and record it as the point set *M*.
2. Select any point $p_1(x_1,y_1)$ in *M*, and calculate the center coordinate based on the coordinates of these two points and *r*. Among them, $p_2(x_2,y_2)$ and $p_3(x_3,y_3)$ are the coordinates of the center of the circle when passing through the two points *p* and $p_1$ and the radius is *r*. The calculation formulas are:

2 $$\begin{aligned} x_{2}= & {} x+\frac{1}{2}\left( x_{1}-x\right) -H \times \left( y_{1}-y\right) \end{aligned}$$

3 $$\begin{aligned} y_{2}= & {} y+\frac{1}{2}\left( y_{1}-y\right) -H \times \left( x-x_{1}\right) \end{aligned}$$

4 $$\begin{aligned} x_{3}= & {} x+\frac{1}{2}\left( x_{1}-x\right) +H \times \left( y_{1}-y\right) \end{aligned}$$

5 $$\begin{aligned} y_{3}= & {} x+\frac{1}{2}\left( y_{1}-y\right) +H \times \left( x-x_{1}\right) \end{aligned}$$

6 $$\begin{aligned} H= & {} \sqrt{\frac{\alpha ^{2}}{S^{2}}-\frac{1}{4}} \end{aligned}$$

7 $$\begin{aligned} S^{2}= & {} \left( x-x_{1}\right) ^{2}+\left( y-y_{1}\right) ^{2} \end{aligned}$$

**Fig. 5:**
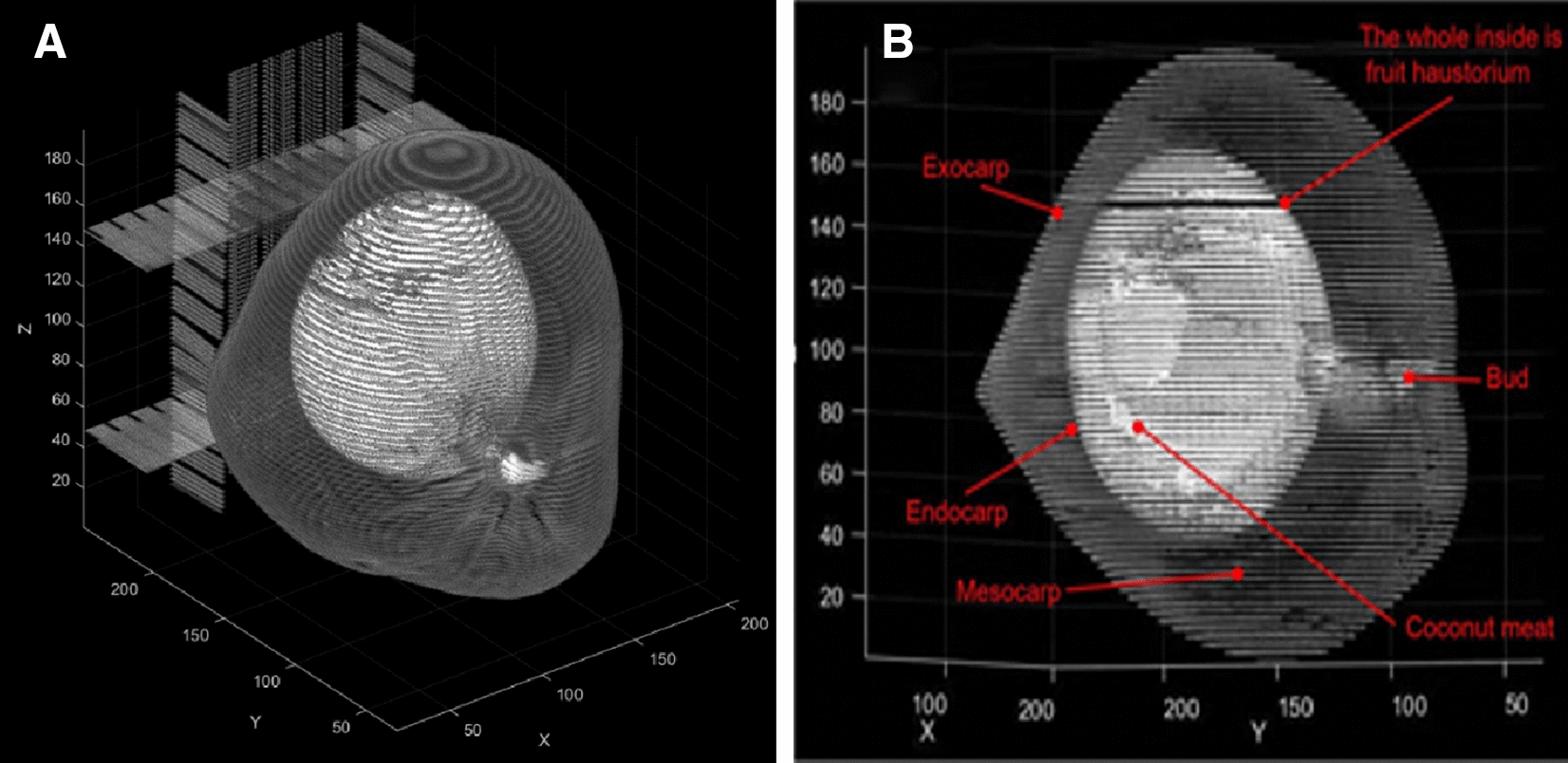
3D model effect and denoising treatment. **A** a 3D point cloud image; **B** the model diagram after denoising and surface fitting

There will be some discrete independent points in the figure, because when the distance is greater than 2*r*, they will be regarded as outliers and will not participate in the rolling process. The model effect of surface fitting can be obtained by this process (Fig. 5b)

### Visualization

Because the point cloud features were composed of discrete points, the model lacked wholeness. We performed a closed meshing operation for surface fitting on the model. The alpha shape rolling ball method [21] was suitable for the feature requirements of the external coconut surface. To find the points that met the requirements, the radius of the rolling ball was set to 0.3 cm, while all external circles corresponding to the connection of points constituted the boundary information; the plot function was then used to draw them, and the color parameters were adjusted to remove the grid lines in order to obtain the model after fitting the discrete points.

**Fig. 6:**
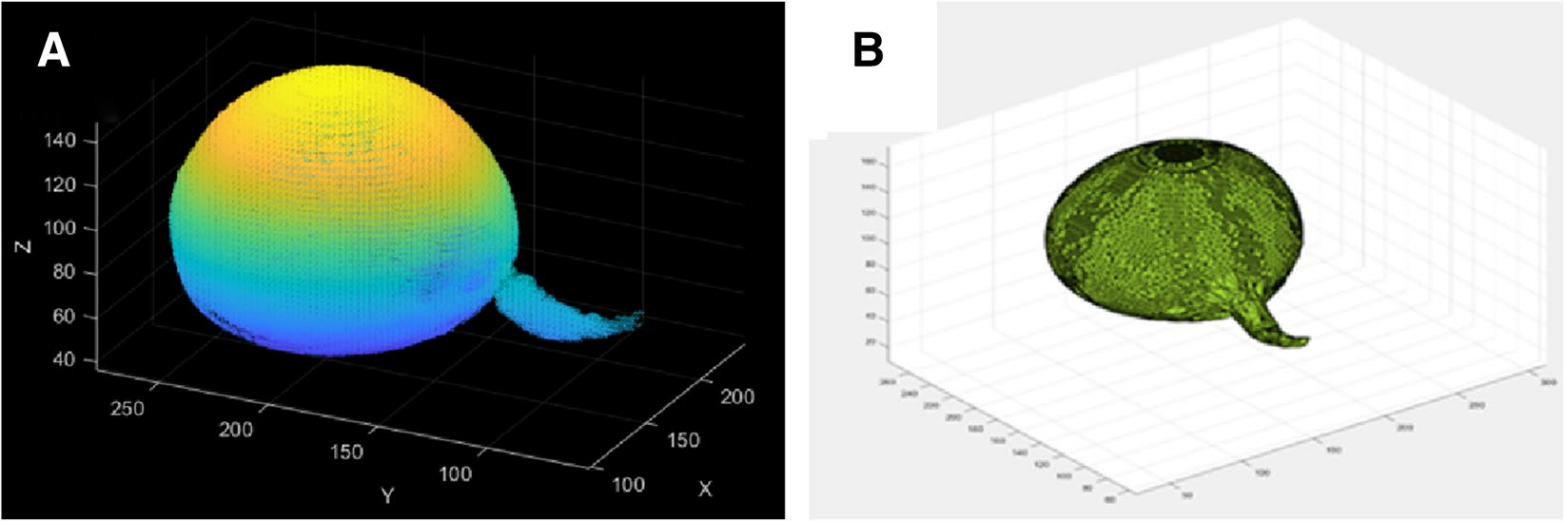
A example of quantitative calculation of organ. **A** Extraction and coloring; **B** the haustorium after meshing

At this point, the basic construction of the model was complete; it was then necessary to quantify it digitally [22]. The model can target any area to obtain its volume. The commonly used fruit haustorium was used as an example for the calculation. The haustorium fruit is extracted according to the RGB value [230,230,230]. Because the target in the color display is not conducive to observation and calculation, we subjected it to color processing (Fig. 6A). Then useed alphashape to perform the grid operation. At this time, the haustorium was composed of small squares (Fig. 6B). Because the 3D coordinates of all points were known, the volume function could be used to obtain the volume value, the models of each coconut fruit in different stages are calculated according to this method [23], the volume value calculated by taking one of the models as an example is 602.9 $\text{cm}^3$.

### Model evaluation

After the above experimental work was completed, we then evaluated the accuracy of the constructed 3D model. We evaluated the model both internally and externally, conducted through in communication with experts at the coconut institute. For the internal factors, we used these four key sets of data as statistical indicators: the long axis of the haustorium fruit, the short axis of the haustorium fruit, the long diameter of coconut water, the short diameter of coconut water. For the external factors, we took the total volume and the triangular mesh number of the reconstructed coconut as the evaluation index. This can take the following form.

#### **Definition 2**

The absolute divergence of coconut is a tuple $\langle H_l, H_s,W_l,W_s,V_{ol},N_{ob}\rangle$, where $H_l$ and $H_s$ represent the statistical difference of long and short axis of haustorium respectively, $W_l$ and $W_s$ represent the statistical difference of long and short diameter of coconut water respectively. And when the error is less than 0.2 cm will be judged to be accurate.$V_{ol}$(volume) represents the volume of the model built, and $N_{ob}$(number of boundary triangle grid) represents the number of triangular meshes on the boundary, which is judged to be accurate as the volume is closer to the reference value and the number of triangular meshes is higher.

### System prototype

**Fig. 7:**
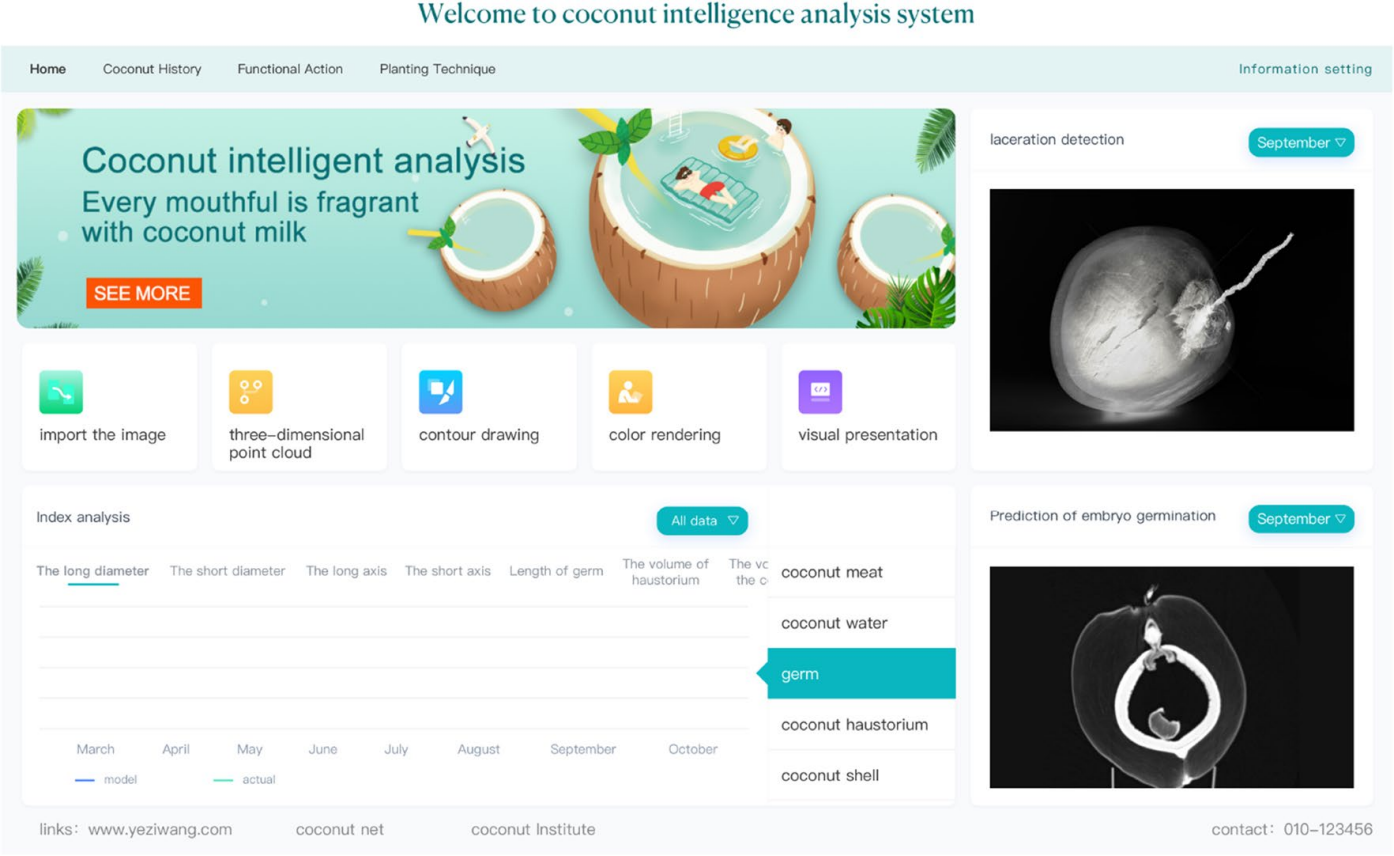
Prototype demonstration of the system

The intelligent coconut analysis system (Fig. 7) is designed according to our working method and the practical application. The Browser-Server architecture is adopted in this system, the development mode is front-end and back-end separated, and the front-end part uses Vue. JS, the development tool is VS Code, and adds the Echarts plug in library. The back-end part uses the Java language, version 1.8, uses the Springboot framework.

When entering the system, followed by the “import the images” function (for uploading coconut CT images), the 3D point cloud (for converting images into the 3D point cloud), contour drawing (for connecting the discrete points of a point cloud map to make it more intuitive), color rendering (applying color according to one’s preferences can help improve the look or highlight important areas), and visual presentation (for outputting the final image after the above steps). Each function block is relatively independent and can be used according to demand.

The reconstructed 3D point cloud display structure is clear at a glance. Through the algorithm, the system can extract the required internal structure and obtain the relevant data. The system provides commonly used structural categories, such as coconut meat, coconut water, germ, coconut haustorium, coconut shell, and so on. After selection, the corresponding length and diameter, long and short axis, volume and other index data can be obtained.

## Result and discussion

In order to prove that the intelligent coconut system can effectively output 3D point cloud models of different coconuts and verify the high accuracy of the 3D model, we performed two experiments by way of illustration. We took 200 coconut fruits and observed them for weeks. Using CT scans, we used CT scanning to obtain a total of 37,950 non-destructive internal growth change maps of various types of coconuts to establish a coconut data set called “CCID”.

**Fig. 8:**
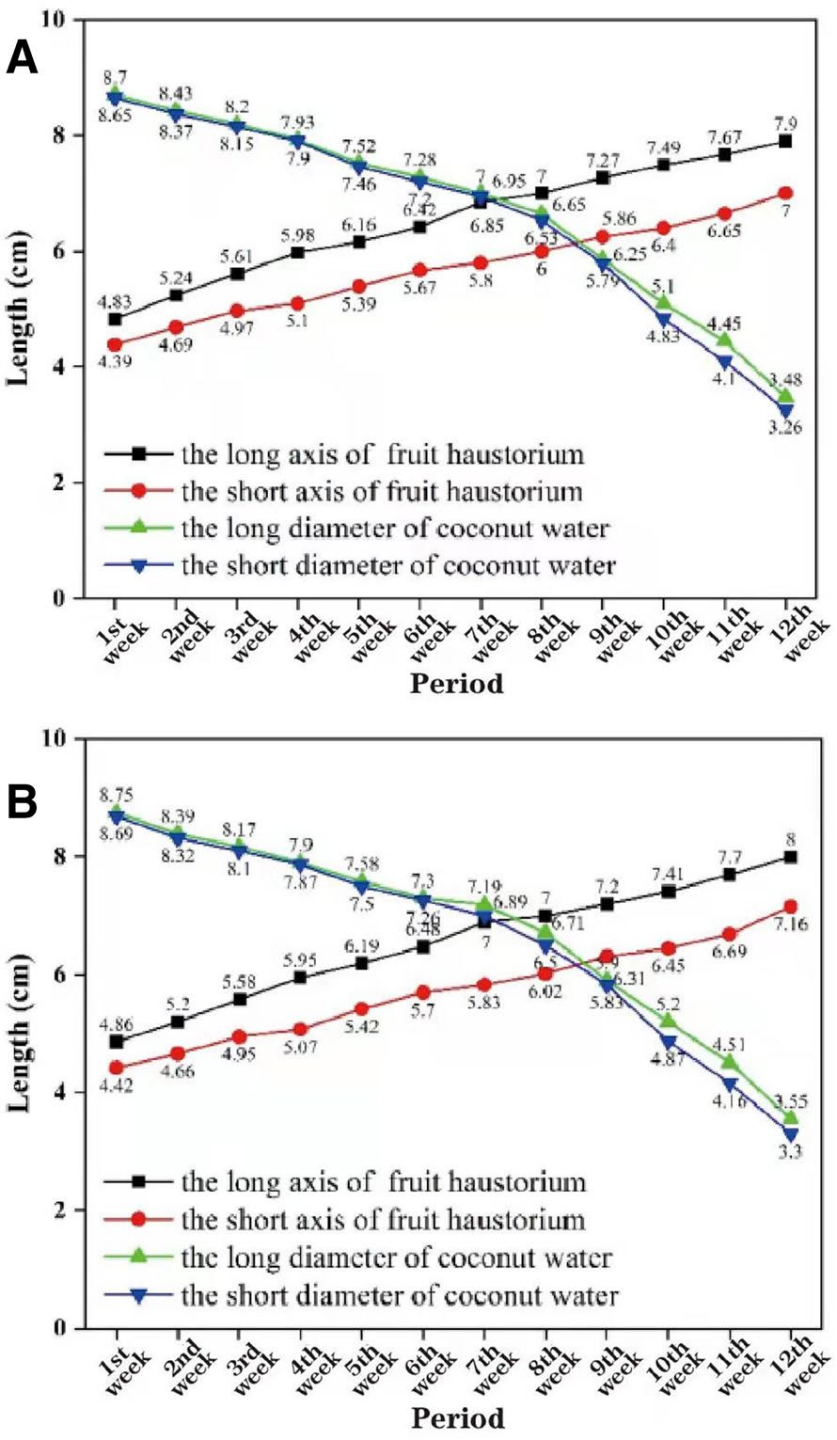
Machine measurements, modeling data (same growth cycle). **A** Important parameter values of coconut fruit in different periods; **B** Corresponding parameter values in the 3D model

We next began our evaluation of internal factors, where the raw parameter values of the actual coconut fruit were measured by the CT machine, and the processed 3D model data thereby generated were measured using the MATLAB platform. Under the same experimental conditions and samples, we recorded the data of both the actual coconut fruit and its 3D model, and the weekly averages of these samples were analyzed and compared as a whole. The horizontal and vertical axes represent the measurement period and the values of each paremeter, respectively (Fig. 8A and B. Figure 8A shows the values of these four factors of the coconut itself measured by the CT machine at different periods. And Fig. 8B shows the values associated with the corresponding 3D model of the coconut at different periods. We observed the coconuts for twelve weeks. These two graphs not only show the growth changes of the internal structure of the coconut, but clearly indicate the small error between the model and the body in each time period. We then calculated their means and variances, further showing how the coconut fruit compared to the corresponding models in terms of important influences over multiple time periods (Table 1). These statistical differences are within the desired interval and strongly indicate the accuracy of our constructed model in terms of internal factors.

**Table 1:** Summary of average and variance comparison results (cm)

Category	Attribute	Long axis of haustorium	Short axis of haustorium	Long diameter of coconut water	Short diameter of coconut water
Coconut fruit	Average	6.535	5.6925	6.7167	6.5992
	Variance	0.90172	5.60087	2.54586	2.82079
3D model	Average	6.5383	5.7233	6.7625	6.6167
	Variance	0.91813	0.65974	2.46632	2.75259

After the evaluation of the internal factors, we addressed the external factors [24]. In our work, two methods for meshing are used to express information, namely Alphashape and Convex Hull [25]. The results were compared to demonstrate which was more suitable.

**Fig. 9:**
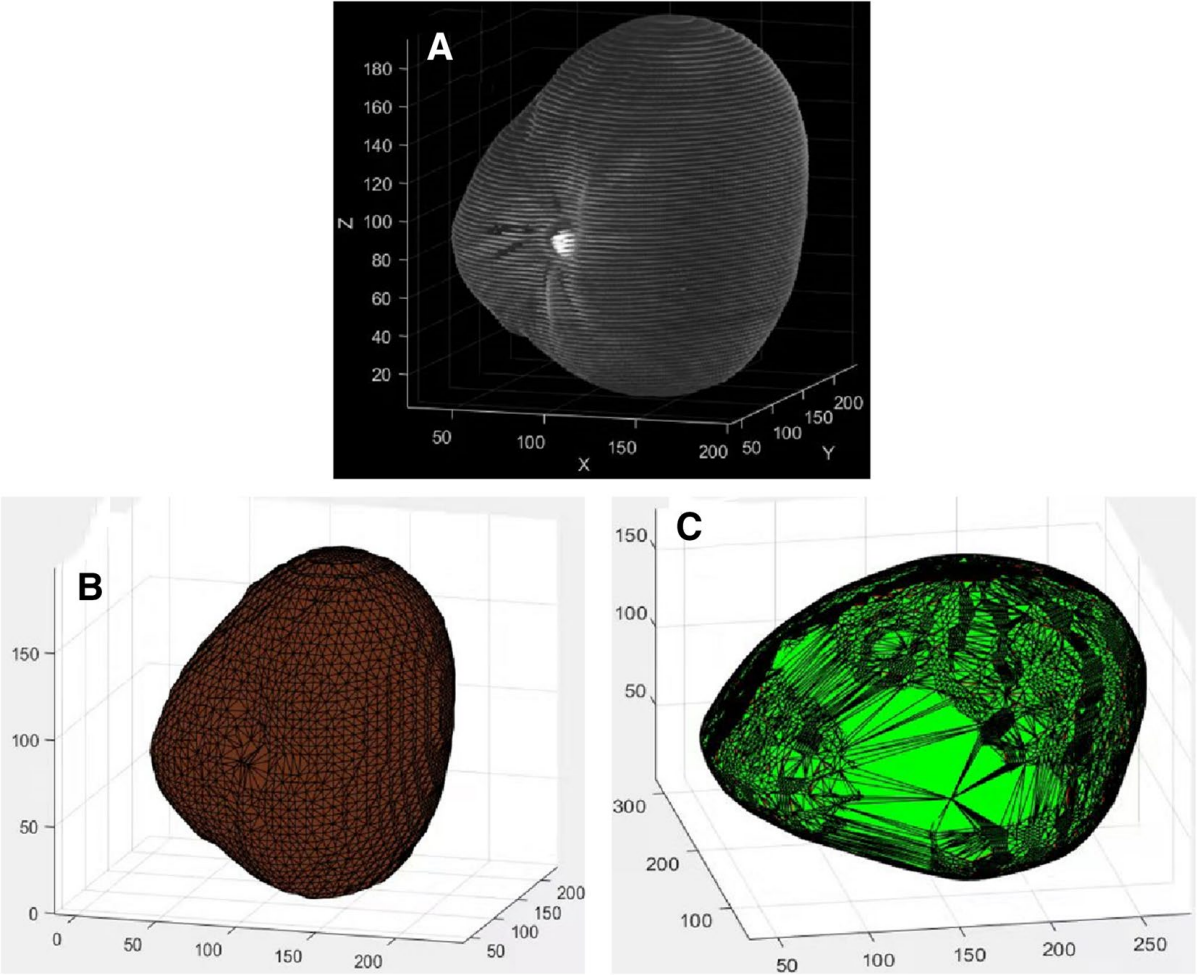
Surface fitting. **A** 3D point cloud of the sample coconut; **B** Alphashape grid; **C** Convex Hull boundary

The mesh reconstruction of the surface is performed using the rolling ball method (Fig. 9B). And we use the volume function in matlab to get its volume and use the numregions function to output the number of triangles in the surface shape, these two indicators are important factors to quantify whether the model is accurate from the outer surface. Correspondingly, we use the Convex Hull to perform surface reconstruction based on the 3D point cloud, the result(Fig. 9C) were obtained from the sample we selected. Then we allowed the program iterate through all the point sets, and based on the number of vertices we can determine whether they were triangles or not, and thus to calculate how many triangles were included, as well as to the volume value as well.

Regarding the comparison between the volume value and the number of boundary grids, we used the data obtained when scanning coconuts on the CT machine as a reference value to compare the final results obtained by the two methods. For the reconstruction of the outer surface, the number of regional grids to a certain extent reflects whether the construction is accurate. Through data comparison, it was found that the volume of the final model formed by the alpha shape method was closer to the reference value, and the number of triangular meshes contained in it was also more than that of the convex hull method. We also introduced the relative error rate. This concept was used to better show the degree of deviation of the results caused by the two methods. The volume value obtained by the alpha shape method was closer to the reference value, and the number of boundary grids was greater, indicating the number of captured points (Table 2). Moreover, the region description was more accurate and the overall error rate was smaller, which further proves the correctness of our selection of the alpha shape method to reconstruct the outer surface. The validity of our method is verified by the above comparison results from the internal structure and the external surface, and the accuracy of our constructed model is proved qualitatively and quantitatively.

**Table 2:** Comparison of the results of outer surface reconstruction between the two methods

Method	Volume($\hbox {cm}^3$)	Number of boundary triangle grid	Relative error of volume (%)
Alphashape	549.03	21038	4.10
Convex Hull	571.58	10934	8.38

Description: The volume reference value of this sample 527.39$\hbox {cm}^3$, the relative error calculation formula is (actual value − reference value)/reference value

## Conclusion

A 3D quantitative imaging model for coconuts was developed, allowing non-destructive observation of the internal development of coconut fruit. The aim was to investigate the internal stratified physiological structure of coconut fruit while identifying its growth pattern through the computation of its relevant tissue and structure data, thereby contributing to coconut cultivation and improvement. The results reveal that the 3D quantitative imaging approach may be utilized to efficiently capture the internal development of coconut fruit. Simultaneously, the 3D point cloud model for coconuts established by this system has high precision, provides a powerful auxiliary function for enhancing coconut cultivation technology, and has a high practical application value in the coconut research sector.

In the future, we hope to utilize the deep learning network to detect cracking inside coconuts and forecast embryo germination. With continuous improvements in technology, imaging instruments and computing power, it is expected that CT can be fully implemented on the sorting line to categorize coconut based on maturity and internal quality. It is anticipated that technological and research advancements will allow for the development of reconstruction techniques and image processing algorithms that will achieve fast and affordable inline CT sorting systems for quality evaluation. We also intend to continuously add new functions to the system. The intelligent system developed herein provides a platform to empower decision-making for researchers within the coconut industry.

## Data Availability

You can contact the first author or corresponding author for coconut CT images and experimental data. Their e-mail addresses are: yuzhang2015@hainanu.edu.cn, hnsuncx@qq.com.
